# Fibroblast Growth Factor 1 Gene-Transfected Adipose-Derived Mesenchymal Stem Cells Modulate Apoptosis And Inflammation In The Chronic Constriction Injury Model of Neuropathic Pain

**DOI:** 10.22037/ijpr.2020.113223.14176

**Published:** 2020

**Authors:** Fatemeh Forouzanfar, Hamid R Sadeghnia, Seyed Javad Hoseini, Ahmad Ghorbani, Hamed Ghazavi, Faezeh Ghasemi, Hossein Hosseinzadeh

**Affiliations:** a *Neuroscience Research Center, Mashhad University of Medical Sciences, Mashhad, Iran. *; b *Department of Neuroscience, Faculty of Medicine, Mashhad University of Medical Sciences, Mashhad, Iran. *; c *Division of Neurocognitive Sciences, Psychiatry and Behavioral Sciences Research Center, Mashhad University of Medical Sciences, Mashhad, Iran. *; d *Department of Medical Biotechnology and Nanotechnology, Faculty of Medicine, Mashhad University of Medical Sciences, Mashhad, Iran. *; e *Pharmacological Research Center of Medicinal Plants, Mashhad University of Medical Sciences, Mashhad, Iran.*; f *Department of Pharmacology, Faculty of Medicine, Mashhad University of Medical Sciences, Mashhad, Iran. *; g *Blood Transfusion Research Center, High Institute for Research and Education in Transfusion Medicine, Tehran, Iran. *; h *Pharmaceutical Research Center, Pharmaceutical Technology Institute, Mashhad University of Medical Sciences, Mashhad, Iran.*; i *Pharmacodynamics and Toxicology Department, School of Pharmacy, Mashhad University of Medical Sciences, Mashhad, Iran.*

**Keywords:** Adipose-derived mesenchymal stem cell, Fibroblast growth factor 1, Transfection, Neuropathic pain

## Abstract

Stem cell therapy is noted for its clinical effect in the treatment of neuropathic pain. This study aimed to investigate the potential anti-apoptotic and anti-inflammatory effects of adipose-derived mesenchymal stem cells (AD-MSCs) and fibroblast growth factor 1 gene-transfected adipose-derived mesenchymal stem cells (AD-MSCs ^FGF1^) on chronic constriction injury (CCI) of the rat’s sciatic nerve. The rats that underwent CCI were treated with AD-MSCs and AD-MSCs ^FGF1^. Bax, Bcl2, and caspases 3, the major contributors of apoptosis, and inflammatory markers including Iba-1, IL1-β, and MMP-2 were evaluated in the lumbar portion (L4-L6) of the spinal cord through western bloating at days 3 and 14. The ratio of Bax/Bcl2, cleaved caspases 3, MMP-2, IL-1β, and Iba1, was elevated in CCI animals compared to sham-operated animals and decreased following treatment with both AD-MSCs and AD-MSCs ^FGF1^. However, the effect of AD-MSCs ^FGF1 ^was significantly higher than AD-MSCs. These data suggest that the administration of AD-MSCs ^FGF1 ^through modulating apoptosis and neuroinflammation could be considered a promising medicine for treating neuropathic pain.

## Introduction

Nerve injury-induced chronic pain was commonly referred to as neuropathic pain. Despite the rapid development of neuroscience associated with drug discovery, due to a lack of precise knowledge of neuropathic pain mechanisms, effective drugs to alleviate neuropathy symptoms are still lacking (1). Evidence suggests that neuropathic pain’s underlying pathophysiology is related to apoptosis and inflammation pathways and involves interactions between neurons, inflammatory immune, and glial cells (2, 3). Cell transplantation is a vibrant research area in the treatment of neuropathic pain (4, 5). Adipose-derived mesenchymal stem cells (AD-MSCs) influence relevant immune modulation and produce an array of neurotrophins and cytokines that positively impact cell viability. Newer studies demonstrated that the combination of growth factors (by genetic manipulation) enhances MSCs’ therapeutic potential (6-9). FGF1 (fibroblast growth factor 1) displays neuroprotective effects (8, 10). It contributes to many physiological processes, such as angiogenesis, neurogenesis, wound healing, and memory functions (10). In the previous study, we transfected AD-MSCs with FGF1 and demonstrated that our ADMSC^FGF1^ could secret FGF1 with proliferative and angiogenic properties (11). ADMSCs^FGF1^ transplantation resulted in a significant decrease in mechanical and thermal hypersensitivity in a rat model of chronic constriction injury (CCI).

Moreover, spinal structural alterations and apoptosis were decreased following AD-MSCs ^FGF1^ administration in CCI rats (12). In light of the benefits found with our previous study, we conducted further examinations on the underlying pathways by which AD-MSCs^FGF1^ exert their actions on neuropathic pain. In the present study, we plan to examine some inflammatory and apoptosis markers’ expressions following administration of AD-MSCs and AD-MSCs^FGF1^ in the spinal cord of the rats submitted to CCI. 

## Experimental

Tris-HCl, ethylene diamine tetraacetic acid (EDTA), β-glycerol phosphate, bromophenol blue, sodium fluoride (NaF), glycerol, tris-buffered saline with tween 20 (TBST), sodium orthovanadate (Na_3_VO_4_), sodium deoxycholate, complete protease inhibitor cocktail (P8340), phenylmethylsulfonyl fluoride (PMSF), sodium dodecyl sulfate (SDS,) and 2-mercaptoethanol (2-ME) were bought from Sigma-Aldrich (St. Louis, MO).

Rabbit polyclonal anti-Bax, rabbit polyclonal anti-Bcl2, anti-cleaved caspase 3, and rabbit polyclonal anti-β-actin antibodies, rabbit horseradish peroxidase-conjugate anti-rabbit IgG or anti-mouse were bought from Cell Signaling Technology (Danvers, MA). Rabbit anti- IL-1β, rabbit anti-MMP2, and mouse monoclonal anti-Iba-1 were purchased from Abcam Technology (Cambridge, UK). High glucose Dulbecco’s modified Eagles medium (DMEM, 4.5 g/L), penicillin and streptomycin, and fetal calf serum (FCS) were bought from Gibco (Carlsbad, CA). Ketamine and xylazine were bought from Alfasan Co (Woerden, Holland). 

*Animals*

Adult male Wistar rats weighing 220-270 g were obtained from the Animal Facility of the Faculty of Medicine, Mashhad University of Medical Sciences, Iran. The animals were kept in a 12 h light-dark cycle environment. Tap water and standard food pellets were available *ad libitum*. All Experiments were done following the National Institutes of Health Guidance for the Care and Use of Laboratory Animals, with the approval of the Animal Ethics Committee of MUMS (#930511).

*Cell cultures*

AD-MSCs were established, characterized, and successfully transfected by a pCMV6-Entry vector with Myc-DDK-tagged ORF clone of *Rattus norvegicus* fibroblast growth factor 1 (rat FGF1), as described previously (11, 13). The AD-MSCs were cultured in DMEM with 10% FBS and 1% antibiotics in a humidified atmosphere of 5% CO_2_, 95% air, 37 °C.

*CCI surgery of sciatic nerve*

Briefly, the rats were anesthetized with a cocktail containing ketamine (64 mg/kg) and xylazine (1.6 mg/kg). The neuropathic pain was induced by performing a chronic constriction injury model on the left sciatic nerve of animals, according to a method previously described by Bennet and Xie (14). The skin of the left thigh’s lateral surface was incised, the sciatic nerve was exposed, and four ligatures of 4-0-gauge chromic catgut were loosely tied around the nerve until a slight twitching was seen in the ipsilateral hind paw. The muscular and skin layer was sutured immediately with silk thread, and the animals were kept in a warm condition until complete recovery. All the surgeries were done by one person.

*Study protocol*

Cell administration was done intravenously on the day of nerve injury, once-daily that continued for two consecutive days. The number of cells and their concentration were 1 × 10^6^ cells/200 µL PBS, chosen according to Forouzanfar *et al*.(12).

*Grouping*

The rats were randomly divided into four groups: Sham-operated rats (which were subjected to surgical procedure without the ligation of the sciatic nerve), CCI rats, CCI + AD-MSCs (CCI rats undergoing the intravenous delivery of AD-MSCs), and CCI + AD-MSCs ^FGF1^ (CCI rats undergoing the intravenous delivery of AD-MSCs ^FGF1^). Sham and CCI-operated rats were intravenously injected with the same amount of PBS alone at the same designated time points. 

*Western blot assay*

The rats were decapitated on days 3, and 14 (n = 6 for each day) and L4-L6 region of spinal cord tissues were rapidly isolated and homogenized in the lysis buffer (10 mM 𝛽-glycerophosphate, 0.2% w/v sodium deoxycholate, 1 mM PMSF, 2 mM EGTA, 50 mM Tris-HCl (pH 7.4), 10 mM NaF, 1 mM sodium orthovanadate (Na_3_VO_4_), 2 mM EDTA, and complete protease inhibitor cocktail). The homogenates were then centrifuged at 10,000 g for 10 min at four ℃. The protein concentrations were assessed by the Bradford assay kit (15). After that, the samples with adjusted protein content were mixed 1:1 with 2 × SDS blue buffer, boiled, aliquoted, and kept at -80 °C. One hundred microgram of each protein extract was separated on 12% sodium dodecyl sulfate-polyacrylamide gel (SDS-PAGE) by electrophoresis and transferred onto PVDF transfer membranes. After transferring, the blots were blocked with 5% skim milk in TBST (20 mM Tris-HCl pH 7.6, 137 mM NaCl, and 0.05% Tween-20) at 4 °C overnight. Then the blots were incubated with primary rabbit anti- IL-1β, rabbit anti-MMP2, mouse monoclonal anti-Iba-1, rabbit polyclonal anti-Bax, rabbit polyclonal anti-Bcl2, rabbit anti-cleaved caspase 3, and rabbit polyclonal anti-β-actin antibodies with an incubation time of about 1-2 h at room temperature. After washing in TBST buffer, the blots were incubated by rabbit horseradish peroxidase-conjugated anti-rabbit IgG or anti-mouse IgG secondary antibody one h at 37 °C. Enhanced chemiluminescence (Pierce, USA) was used to visualize the peroxidase-coated bands and visualized using Alliance 4.7 Gel Doc (UK). Densitometric analysis for specific protein bands was done using NIHe Image J software. The protein levels of each band were normalized against the corresponding B-actin band (12). 

*Statistical analysis*

All behavioral data are expressed as mean ± SEM (standard error of the mean) and analyzed by one-way ANOVA, followed by Tukey-Kramer *post-hoc* test for multiple comparisons. The values of *p* < 0.05 were considered significant.

## Results

*AD-MSC and AD-MSCs* ^FGF1^ *decreased cleaved Caspase-3 and Bax/Bcl-2 expressions in CCI rats’ spinal cord on day 3* **.**

**Figure 1:**
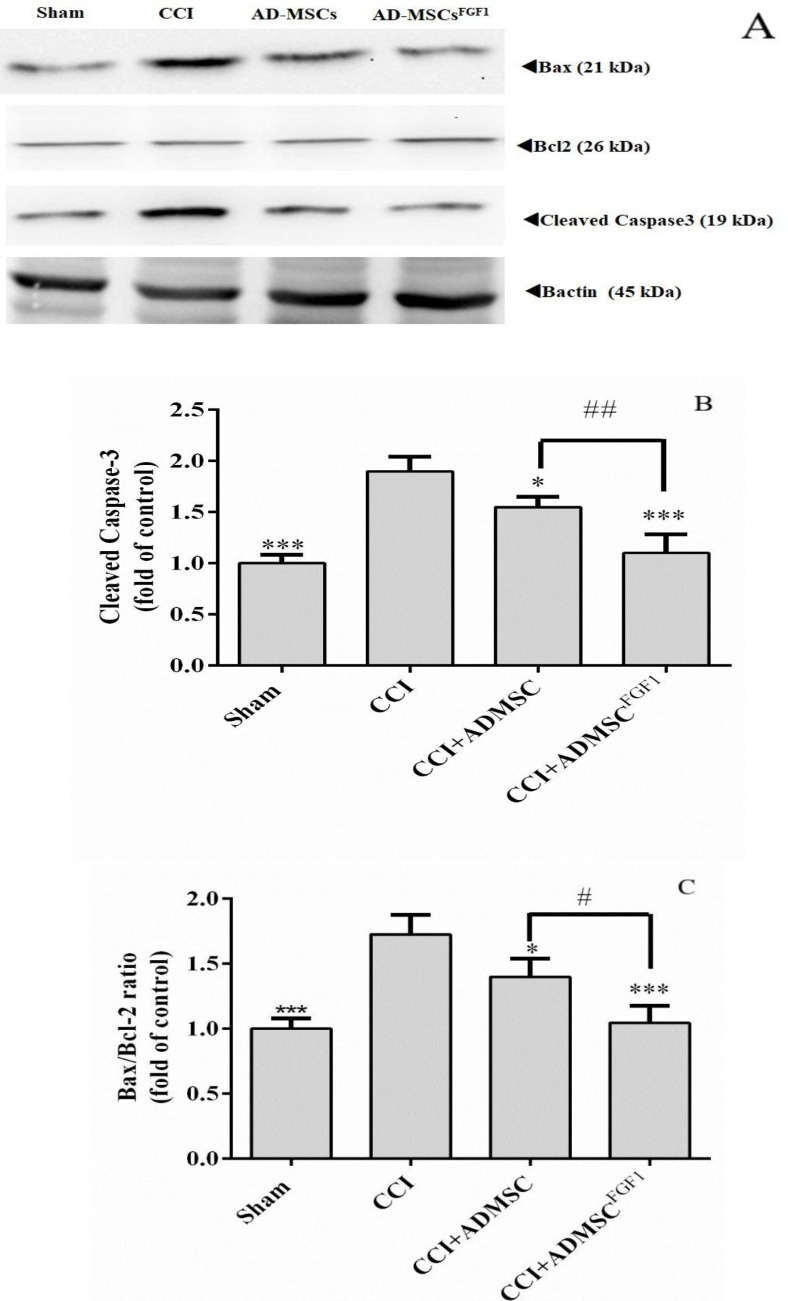
Effects of AD-MSCs and AD-MSCs ^FGF1^ on apoptosis-related protein expressions in L4-L6 dorsal horn of spinal cord of CCI rats on day 3. (A) Representative images of pro-apoptotic (Cleaved Caspase-3 and Bax) and anti-apoptotic (Bcl-2) by western blotting. (B, C) the bar graphs show the relative protein expressions of cleaved caspase-3 and Bax/Bcl-2 ratio, respectively. 𝛽-actin is the loading protein control. Each value represents the mean ± SEM. *p < 0.05, ***p < 0.001 *vs*. CCI group; #*p *< 0.05, ##*p *< 0.01 *vs*. AD-MSCs group. (n = 6)

As indicated in (Figures 1A-C), CCI led to a substantial increase in the expression of cleaved Caspase-3 (1.9-fold) and Bax/Bcl-2 ratio (1.7-fold), as compared with the sham group (*p *< 0.001). On the contrary, AD-MSCs and AD-MSCs^FGF1^ were able to diminish the Caspase-3 expression to 1.6 (*p* < 0.05) and 1.1 (*p *< 0.001) fold in CCI rats, respectively. This corrective effect was more significant in the CCI + AD-MSCs^FGF1 ^group (*p* < 0.01 compared to the CCI + AD-MSCs group).

Also, AD-MSCs and AD-MSCs^FGF1^ administration resulted in a decrement of Bax/Bcl-2 expression to 1.4 (*p *< 0.05) and 1 (*p* < 0.001) fold in CCI rats, respectively. The CCI + AD-MSCs^FGF1 ^group exhibited a signiﬁcantly lower expression than the CCI + AD-MSCs group (*P* < 0.05).

*AD-MSC and AD-MSCs* ^FGF1^ *decreased IL-1β expression in the spinal cord of CCI rats on day 14*

**Figure 2:**
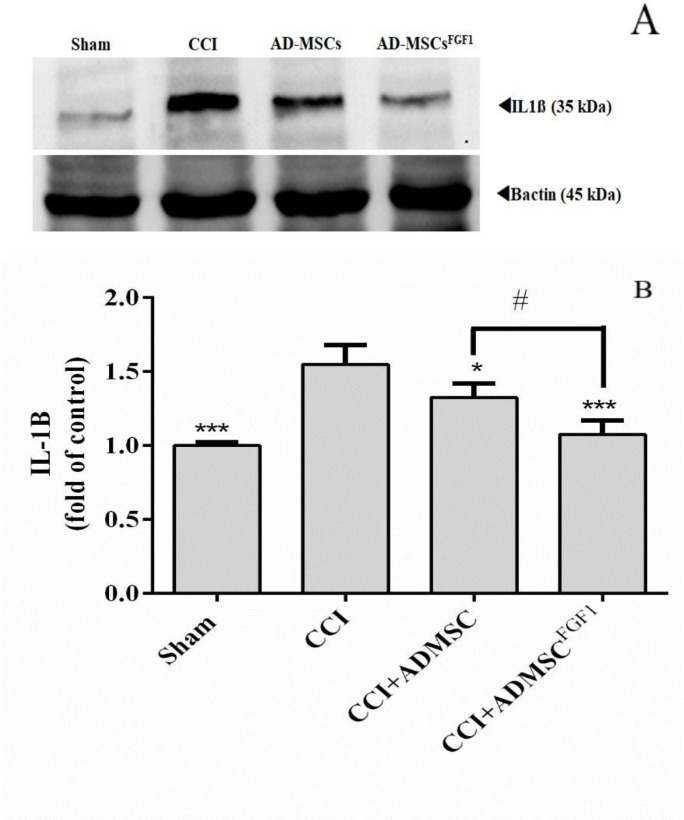
Effects of AD-MSCs and AD-MSCs ^FGF1^ on expressions of IL-1β protein in L4-L6 dorsal horn spinal cord of CCI rats on day 14. (A) Representative images of IL-1β by western blotting. (B) Tthe bar graphs show the relative protein band expressions of IL-1β. 𝛽-actin is the loading of protein control. Each value represents the mean ± SEM. *p < 0.05, ***p < 0.001 *vs*. CCI group; #*p *< 0.05 *vs*. AD-MSCs group. (n = 6).

As shown in (Figure 2), there was a significantly higher relative density of IL-1β expression in CCI rats (1.5 fold of control), in comparison to the sham-operated animals (*p* < 0.001). On the other hand, AD-MSCs and AD-MSCs^FGF1^ were able to decrease the IL-1β expression to 1.3 (*p *< 0.05) and 1.1 (*p* < 0.001) fold in CCI rats, respectively. Furthermore, the effect was more significant in the CCI + AD-MSCs^FGF1 ^group (*p* < 0.05 compared to the CCI + AD-MSCs group).

*AD-MSC and AD-MSC* ^FGF1^ *decreased Iba-1 expression in the spinal cord of CCI rats on day 14.*

**Figure 3:**
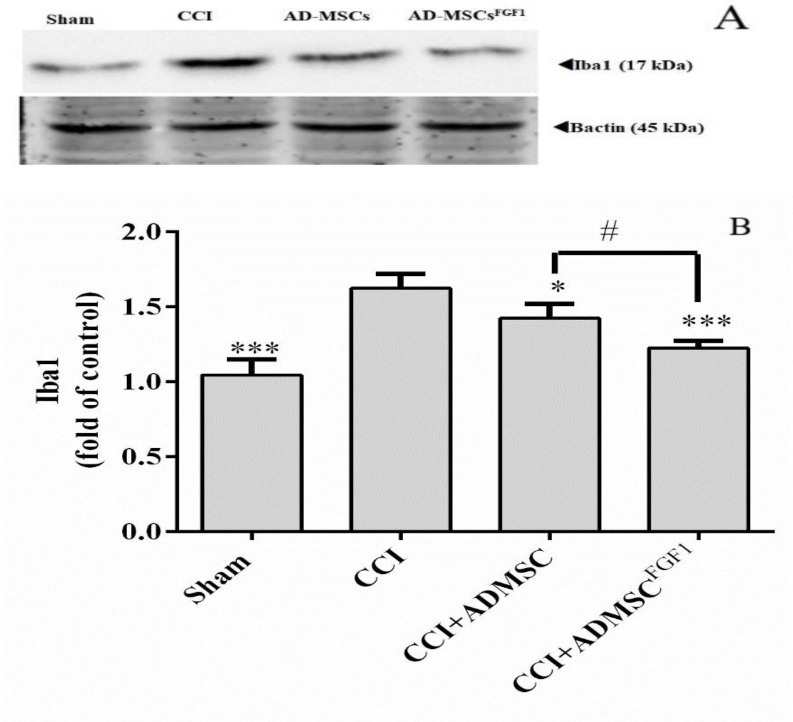
Effects of AD-MSCs and AD-MSCs ^FGF1^ on expressions of Iba-1 protein in L4-L6 dorsal horn spinal cord of CCI rats on day 14. (A) Representative images of Iba-1 by western blotting. (B) The bar graphs show the relative protein band expressions of Iba-1. 𝛽-actin is the loading of protein control. Each value represents the mean ± SEM. *p < 0.05, ***p < 0.001 *vs*. CCI group; #*p *< 0.05 *vs*. AD-MSCs group. (n = 6)

As illustrated in (Figure 3), Iba-1 expression was increased in CCI rats (1.6 fold) as compared with the sham-operated animals (*p* < 0.001). On the contrary, AD-MSCs and AD-MSCs^FGF1^ administration resulted in a decrement of Iba-1 expression to 1.4 (*p* < 0.05) and 1.2 (*p *< 0.001) fold in CCI rats, respectively. The CCI + AD-MSCs^FGF1 ^group showed a signiﬁcantly lower expression than the CCI + AD-MSCs group (*p* < 0.05).

*AD-MSC and AD-MSCs* ^FGF1^ *decreased MMP-2 expression in the spinal cord of CCI rats on day 14.*

**Figure 4.**
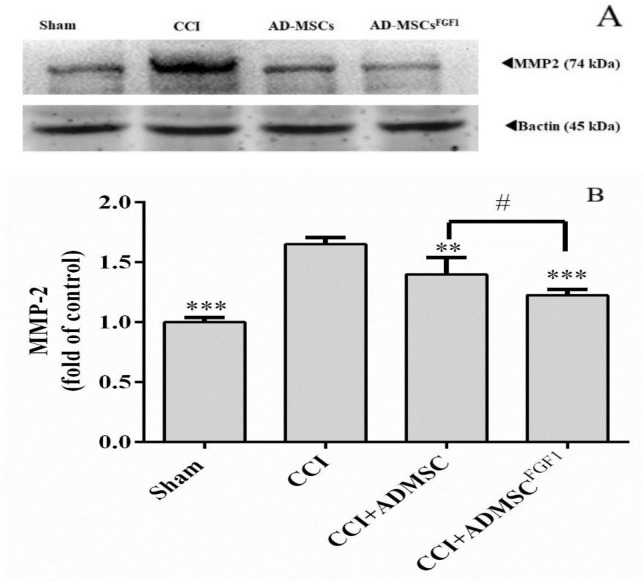
Effects of AD-MSCs and AD-MSCs ^FGF1^ on expressions of MMP-2 protein in L4-L6 dorsal horn spinal cord of CCI rats on day 14. (A) Representative images of MMP-2 by western blotting. (B) The bar graphs show the relative protein band expressions of MMP-2 (B). 𝛽-actin is the loading of protein control. Each value represents the mean ± SEM. **p < 0.01, ***p < 0.001 *vs*. CCI group; #*p *< 0.05 *vs*. AD-MSCs group. (n = 6)

As shown in (Figure 4), western blot analysis in the CCI group revealed a significant increase in the MMP-2 expression (1.7 fold) in comparison to the sham-operated animals (*p* < 0.001). On the other hand, AD-MSCs and AD-MSCs^FGF1^ were able to decrease the MMP-2 expression to 1.4 (*p* < 0.01) and 1.2 (*p* < 0.001) fold in CCI rats, respectively. The effect was more significant in the CCI + AD-MSCs^FGF1 ^group (*p* < 0.05 compared to the CCI + AD-MSCs group). 

## Discussion

In the present study, we demonstrated that the intravenous administration of AD-MSCs ^FGF1^ effectively diminishes apoptosis and inflammation of neuropathic pain that originates from a peripheral lesion. Following the peripheral nerve injury, a cascade of neuroinflammation-related events occurs in pain generation (3, 16). Microglia are one of the first spinal cord cell types activated within the first hours of peripheral nerve injury, which continues for at least numerous months in experimental neuropathies. Microglial activation is characterized by the expression of Iba1 (3). The release of IL-1β from spinal cells was enhanced following the responses to pathophysiological changes during neuropathic pain (17). More pertinently in spinal cord neurons, IL-1β enhanced excitatory AMPA and NMDA-induced currents whilst suppressed GABA- and glycine-induced inhibitory currents (18). Previous studies suggested that IL-1β induction of NMDA-currents may be via PK-C that phosphorylates NMDA subunits, NR1 and NR2B (19). The inhibition of IL-1β signaling prevented transcriptional up-regulation of the COX-2 gene, which resulted in the reduction of mechanical hyperalgesia and normalization of pain sensitivity (20). Matrix metalloproteinases (MMPs) are zinc-dependent proteins, have imperative roles in numerous proteolytic reactions, and are associated with different neurodegenerative disorders (21). They degrade structural proteins of the extracellular and increase the amount of pro-inflammatory cytokines such as IL-1β (22). In the context of neuropathic pain, elevated MMP-2 contributes to the development of neuropathic pain (23). In accordance with previous literature (21, 24), Iba1, IL-1β, and MMP-2 upregulation were seen on day 14 post-CCI. Administration of AD-MSCs and AD-MSCs ^FGF1^ significantly attenuated the contents of Iba1, IL-1β, and MMP-2, in the spinal cord of animals subjected to nerve injury. The corrective effect was greater in the AD-MSCs ^FGF1^ group. AD-MSCs are known to provide anti-inﬂammatory and anti-apoptotic by producing many growth factors, cytokines, and chemokines (25). In a study by Sacerdote’s *et al*., AD-MSc administration reduced the pro-inflammatory cytokine IL-1β. It increased the anti-inflammatory cytokine IL-10 in the injured nerve of the CCI rats. Besides, AD-MSc administration decreased the expression of inducible nitric oxide synthase in CCI animals’ spinal cord (26). In a study conducted by Siniscalco *et al*., injection of human MSCs to neuropathic mice led to decreased NP-like behaviors, mRNA levels of the pro-inﬂammatory interleukin IL-1β mouse gene, as well as astrocytic and microglial cell activations (27). FGF1 induced the expression of T helper type 2 (T_h2_) cytokine IL-4 and sequential upregulation of arginase-I** (**Arg I) (28). Arg I enhances the synthesis of polyamines that promote axonal regeneration and prevent cell death after injury (29). Subsequently, the upregulation of Arg I leads to a decrement of the inflammatory response and neuropathic pain occurrence (30). In a study conducted by Lin *et al*., following cervical root transection, intercostal nerve grafts and FGF1 resulted in a decrement of microglial and IL-1β-positive astrocyte reactions in the spinal cord, along with a signiﬁcant increase in arginase I expression (30). The apoptotic processes appear and develop during the first few days after the induction of CCI. Subsequently, increased expression of anti-apoptotic Bcl-2 family genes may inhibit more neuronal loss. It could be associated with the own neuroprotection mechanisms of the nervous system (31, 32). The results presented here show a significant elevation in the Bax/Bcl2 ratio and cleaved caspases 3 in CCI animals on day three post-surgery. Treatment with AD-MSCs and AD-MSCs ^FGF1 ^leads to correction of the expressions of these factors. Again, the corrective effect was greater in the AD-MSCs ^FGF1^ group. Previously, we also showed that FGF1 therapy, along with stem cell transplantation, markedly diminished the CCI-induced DNA fragmentation and apoptosis in the spinal cord of CCI rats (12). FGF1 is a differentiation and survival factor with multiple biological effects, such as angiogenesis, mitogenesis, and repair. It is highly expressed in the central and peripheral nervous systems (33, 34). FGF1 is a repressed target gene of p53. The overexpression of FGF1 via increasing MDM2 (mouse double minute 2) expression leads to a decrement of pro-apoptotic and the anti-proliferative effects of p53 and Bax, a pro-apoptotic protein (35). In the presence of DNA damages, FGF1 leads to a decrement in p53 stability activities and p53-dependent transactivation of the pro-apoptotic genes, such as puma, noxa, and caspases 3 (34). In conclusion, the present study demonstrated that intravenous administration of AD-MSCs, transfected with the FGF1 gene, resulted in significant improvements in the spinal cord’s apoptosis and inflammation in a rat model of CCI, with greater efficacy than non-modified AD-MSCs and has vast potential for future studies.
